# Effectiveness of the SYNCHRONIZE + Brief Intervention in Improving Mediterranean Diet Adherence, Nutritional Quality and Intake Pattern in Persons with Fibromyalgia and Chronic Fatigue Syndrome

**DOI:** 10.3390/nu17010011

**Published:** 2024-12-24

**Authors:** Noèlia Carrasco-Querol, Lorena Cabricano-Canga, Nerea Bueno Hernández, Carme Martín-Borràs, Alessandra Queiroga Gonçalves, Anna Vila-Martí, Blanca Ribot, Judit Solà, Carme Valls-Llobet, Rosa Caballol Angelats, Pilar Montesó-Curto, Elisabet Castro Blanco, Macarena Pozo Ariza, Sandra Carreres Rey, Laura Pla Pagà, Mònica Dearos Sanchís, José Fernández-Sáez, M. Rosa Dalmau Llorca, Carina Aguilar Martín

**Affiliations:** 1 Unitat de Suport a la Recerca Terres de l’Ebre, Fundació Institut Universitari per a la Recerca a l’Atenció Primària de Salut Jordi Gol I Gurina (IDIAPJGol), 43500 Tortosa, Spain; nbueno@idiapjgol.org (N.B.H.); aqueiroga@idiapjgol.info (A.Q.G.); ecastro@idiapjgol.info (E.C.B.); mpozo@idiapjgol.org (M.P.A.); jfernandez@idiapjgol.info (J.F.-S.); caguilar.ebre.ics@gencat.cat (C.A.M.); 2 EAP Dreta Eixample, CAP Roger de Flor, C/Roger de Flor 194, 08013 Barcelona, Spain; lcabricano@eapdretaeixample.cat; 3 Departament de Bioquímica i Biotecnologia, Facultat de Medicina i Ciències de la Salut, Universitat Rovira i Virgili (URV), 43201 Reus, Spain; 4 Servei d’Atenció Primària Terres de l’Ebre, Institut Català de la Salut (ICS), 43500 Tortosa, Spain; rcaballol.ebre.ics@gencat.cat (R.C.A.); mpmonteso.ebre.ics@gencat.cat (P.M.-C.); scarreres.ebre.ics@gencat.cat (S.C.R.); lplap.ebre.ics@gencat.cat (L.P.P.); rdalmau.ebre.ics@gencat.cat (M.R.D.L.); 5 Departament d’Activitat Física i Fisioteràpia, EUSES Terres de l’Ebre, Universitat Rovira i Virgili (URV), 43500 Tortosa, Spain; 6 Red de Investigación en Cronicidad, Atención Primaria y Promoción de la Salud (RICAPPS), 08007 Barcelona, Spain; 7 Research Group M3O—Methodology, Methods, Models and Outcomes, Departament Ciències de la Salut Bàsiques, Facultat de Ciències de la Salut i el Benestar, Centre for Health and Social Care Research (CESS), Universitat de Vic-Universitat Central de Catalunya (UVic-UCC), 08500 Vic, Spain; anna.vilamarti@uvic.cat (A.V.-M.); blanca.ribot@uvic.cat (B.R.); judit.sola@uvic.cat (J.S.); 8 Centro de Analisis y Programas Sanitarios (CAPS), 08010 Barcelona, Spain; carmevallsllobet@hotmail.com; 9 Fundació Institut Universitari per a la Recerca a l’Atenció Primària de Salut Jordi Gol I Gurina (IDIAPJGol), 08007 Barcelona, Spain; 10 Departament de Medicina i Cirurgia, Facultat de Medicina i Ciències de la Salut, Universitat Rovira i Virgili (URV), 43201 Reus, Spain; 11 Unitat d’Endocrinologia i Nutrició, Hospital de Tortosa Verge de la Cinta, Institut Català de la Salut (ICS), 43500 Tortosa, Spain; mdearos.ebre.ics@gencat.cat; 12 Institut d’Investigació Sanitària Pere Virgili (IISPV), Hospital de Tortosa Verge de la Cinta, Institut Català de la Salut (ICS), 43500 Tortosa, Spain; 13 Departament d’Infermeria, Facultat d’Infermeria, Universitat Rovira i Virgili (URV), Campus Terres de l’Ebre, 43500 Tortosa, Spain; 14 Unitat d’Avaluació i Recerca, Direcció d’Atenció Primària Terres de l’Ebre i Gerència Territorial Terres de l’Ebre, Institut Català de la Salut (ICS), 43500 Tortosa, Spain

**Keywords:** nutritional intervention, fibromyalgia, mediterranean diet, chronic fatigue syndrome, nutritional quality, dietary patterns

## Abstract

Background: Multidisciplinary lifestyle interventions are being researched to treat fibromyalgia. However, the impact of nutrition as a key treatment component is little studied. This study aimed to evaluate the effectiveness of the SYNCHRONIZE + lifestyle multidisciplinary intervention in improving adherence to the Mediterranean diet, nutrition quality and dietary intake pattern in persons with fibromyalgia and chronic fatigue syndrome. Methods: A pragmatic randomized clinical trial was conducted in primary care. Data were collected using the 17-item energy-restricted Mediterranean Adherence Screener (er-MEDAS), the food frequency questionnaire (sFFQ) and the 24 h recall questionnaire (24 HR), in addition to chrono-nutritional, anthropometric, and body composition data, at baseline and 3-, 6-, and 12- month follow-up visits, and statistically analyzed. Results: A total of 158 participants were evaluated. Results showed the effectiveness of the intervention in improving adherence to the Mediterranean diet. The adherence depended on the group-time interaction being positive and significant at 3 and 6 months post-intervention in the INT group and on the participant age and educational level. Specifically, the intake of legumes, fruits, vegetables, nuts and blue fish was increased, while the intake of sweets and pastries, butter and cream and red and processed meat was reduced. Furthermore, the intake of chips and candies was also reduced, and the consumption of fermented food (yogurts, cheese, kefir) increased. Thus, general diet quality improved. Interestingly, the intake of key nutrients such as protein and iron increased. Furthermore, the number of night eaters was decreased significantly. Muscle mass index was also improved in the intervention group. These results were maintained in the medium to long term. Conclusion: SYNCHRONIZE + is a brief, low-cost, multidisciplinary intervention effective in improving adherence to the Mediterranean diet and improving nutritional and dietary intake patterns in persons with fibromyalgia and chronic fatigue syndrome. Further evaluation of the effect on quality of life and symptoms is needed.

## 1. Introduction

Lifestyle interventions, mainly based on nutrition, physical activity and chronobiology have shown an enormous potential for preventing and managing a significant number of chronic diseases. A growing body of evidence points to the effectiveness of lifestyle interventions in preventing and managing chronic diseases, including cardiovascular disease, as reported by the PREDIMED and PREDIMED-Plus projects, among other studies. Adherence to and interventions based on the Mediterranean diet were associated with a lower risk of hepatic steatosis, stroke, total and cause-specific mortality, central obesity and hyperglycemia. Additionally, they showed benefits in reducing fractures related to osteoporosis in people with cardiovascular risk, reducing the death rate from breast cancer, lowering blood pressure in hypertensive patients and improving cognitive health [1,2,3,4,5,6,7,8,9,10]. Along these lines, diet quality has also been associated with mental health outcomes, including depression, anxiety, stress levels and dementia [11]. Furthermore, lifestyle interventions including nutrition, physical activity and sleep quality, among other components, were effective in improving quality of life in people with multiple sclerosis, a complex neurodegenerative autoimmune disease [12]. Increasing knowledge about the clinical impact of such lifestyle interventions is crucial to opening the door to new and complementary therapeutical interventions that can help improve quality of life and disease management.

Fibromyalgia (FM) has a complex, unknown etiology and is difficult to manage. It is considered a central sensitization syndrome, potentially involving neurological, metabolic, inflammatory and immunological mechanisms, as well as cognitive and emotional factors. Among these mechanisms, it has been postulated that the hyperactivation of the brain areas that modulate pain results in an altered pain perception. Furthermore, the role of neuroinflammation and the immune system is currently under study, pointing to links with autoimmunity [13].

The main symptoms of FM are chronic diffuse pain, insomnia, chronic fatigue, and gastrointestinal problems. Those affected are mostly women (95%) [14]. The current world-wide prevalence is 0.72–3.7% [15]. People with FM are also often affected by chronic fatigue syndrome (CFS). This is another central sensitization syndrome and autoimmune disease that is not completely understood, which has a huge impact on the quality of life of persons with FM and associated CFS.

Little is currently known about the origin, causes, and mechanisms that lead to the development of FM. The triggering factors may be the combination of genetic predisposition and environmental factors, such as significant acute or chronic psychological stress, physical overload, chrono-disruption, nutritional deficiencies and exposure to environmental toxins or pathogens, among others [16]. FM manifests in flares that involve pain exacerbation, fatigue and discomfort, hugely affecting quality of life. Therapeutic objectives currently focus on reducing the sensation of pain and improving mental health, which is usually affected as well. Traditionally, FM has been approached through pharmacological treatment, including tricyclic antidepressants, analgesics, opiates and anticonvulsants, among others. The expected improvements are often not achieved, and patients suffer from potential secondary effects. Current clinical guides recommend against NSAIDs, strong opioids and benzodiazepines due to their side effects. There is no good evidence to justify the association of several drugs [17]. However, the guidelines do recommend patient education interventions [18]. Non-pharmacological multicomponent interventions based mainly on health education, cognitive behavioral therapy and physical activity have recently been developed and evaluated, yielding better results in improving the main symptoms and quality of life [19,20].

The potential of including nutrition and chronobiology in multidisciplinary interventions for FM and CFS has been also reported and thoroughly discussed [21]. However, they are currently not usually the key components in interventions, and clinical evidence is still scarce. A nutrition-based strategy aimed at improving nutritional quality, food intake pattern and adherence to the Mediterranean diet or other healthy diets, as well as increasing the intake of fiber and antioxidant and anti-inflammatory foods could help. Such a strategy would optimize the intake of macro- and micronutrients to prevent deficiencies, modulate oxidative stress and inflammation and improve gut microbiota and immune health through different mechanisms, leading to significant health benefits. Likewise, improving intake timing patterns, the number of meals per day and night fasting may lead to benefits through similar mechanisms [22].

In light of all this, and in search of new therapeutic multidisciplinary approaches addressing these objectives, there is an ongoing study looking into the health benefits for people with FM of a brief multicomponent intervention based on nutrition, chronobiology and physical activity, known as SYNCHRONIZE + [23]. As a key first step, the objective of the present study was to thoroughly evaluate the effectiveness of the intervention in improving lifestyle outcomes, such as food intake (food intake pattern, nutritional quality and adherence to the Mediterranean diet) and chronobiological aspects (number of meals a day and night fasting). This study is essential to understand whether the intervention was effective in improving lifestyle factors and to consider those results in further SYNCHRONIZE + studies.

## 2. Materials and Methods

### 2.1. Study Design and Objective

A randomized clinical trial (NCT05719493) was conducted to evaluate the SYNCHRONIZE + multicomponent group intervention based on nutrition, chronobiology and physical activity to improve quality of life in persons with FM, often with CFS as a comorbidity, from southern Catalonia [23]. Intervention and data collection were carried out between October 2021 and May 2024. The first step and the specific aim of this study was to evaluate the effectiveness of the SYNCHRONIZE + intervention in improving lifestyle outcomes. Thus, intermediate outcomes related to adherence to an energy-restricted Mediterranean diet, nutritional quality and intake pattern, as well as chrono-nutrition outcomes, anthropometric measures and body composition were studied. The Clinical Research Ethics Committee of the Jordi Gol Primary Care Research Institute approved the study (codes: 21/154-P and 22/087-P), which was conducted in accordance with the Declarations of Helsinki and Tokyo. 

### 2.2. Study Population 

The study participants were primary care patients of the Catalan Health Institute (ICS) of Terres de l’Ebre (South Catalonia). They were diagnosed with FM, with or without CFS (International Classification of Diseases-10, M79.7 and G93.3, respectively) [24]. Inclusion and exclusion criteria were applied following the original protocol [23]. Eligibility criteria included people aged 18–65 with a recent diagnosis (<10 years) of FM, with or without CFS. FM and CFS diagnosis were based on current criteria [25,26]. Study information was provided to participants, and written informed consent was obtained before the study. 

### 2.3. SYNCHRONIZE + Intervention

SYNCHRONIZE + is a brief multicomponent group-based education intervention developed to provide key lifestyle information (about nutrition, chronobiology and physical activity) and encouragement to people diagnosed with FM, with or without CFS. Four group sessions were held over 2 consecutive weeks for a total of 12 h (2 sessions/week, 3 h/session) at the primary healthcare center (PHC). More details are available at Carrasco-Querol et al. (2023) [23].

**Figure 1:**
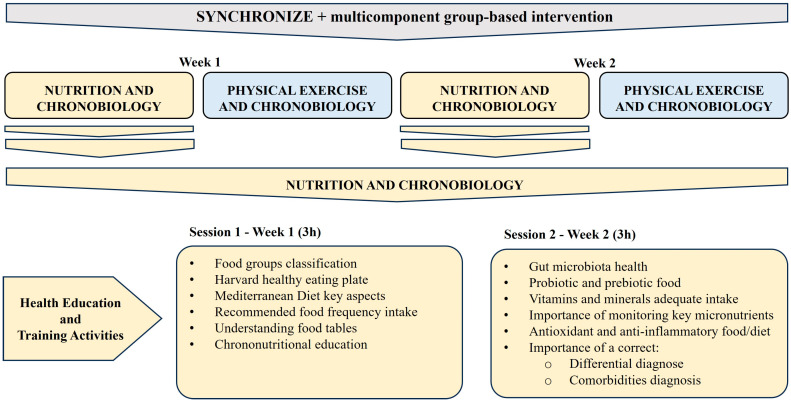
Intervention details on nutrition and chronobiology.

Nutrition sessions: Two sessions lasting 3 h each (6 h in total) focused on nutrition (Figure 1). Session 1 included education and training activities on food groups, the Harvard healthy eating plate, key aspects to following a Mediterranean diet, recommended frequency of food intake, reading and understanding food labels and chrono-nutrition (meals/day, meal timing, night fasting, etc.). Session 2 included information on the importance of the following aspects to improve health outcomes and quality of life: the health of the gut microbiota and the intake of probiotic and prebiotic food, adequate intake of vitamins and minerals to prevent deficiencies, food combinations to promote micronutrient absorption, the monitoring of key micronutrients (ferritin, vitamin B12, vitamin D, etc.) by healthcare professionals in the case of FM and CFS, a diet rich in antioxidant and anti-inflammatory nutrients and with limited pro-inflammatory nutrients, a correct differential diagnosis and concomitant comorbidities (altered thyroid metabolism, digestive problems etc.). Participants received a fridge magnet and leaflet with key information to take home, developed specifically for the project. Furthermore, they could share photos with examples of their home-cooked meals in a voluntary WhatsApp group to comment on them and improve nutritional quality. The control group continued with care as usual and had an individual visit with an FM specialist who informed patients of the disease using a leaflet. Participants in the control group were offered the opportunity to participate in the SYNCHRONIZE + group at the end of the study.

### 2.4. Data Collection and Outcomes

Study participants were recruited, randomized and allocated to the intervention (INT group) or control groups (CTR group). Participants were sequentially allocated to a study group according to the randomization list, following Efron’s procedure [27]. They were informed about their group allocation and provided with the schedule for their assigned sessions or visits [23]. 

Data were collected at the baseline visit and at 3, 6 and 12 months post-intervention. At each follow-up visit, data related to nutrition, physical activity, chronobiology and health outcomes were collected. Regarding nutrition, food intake data were collected from the 17-item energy-restricted Mediterranean Diet Adherence Screener (er-MEDAS) [28] and are considered the main outcome of the present study. Secondary outcomes were assessed with the short food frequency questionnaire (sFFQ) [29], the 24 h dietary recall (24 HR) [30] and chrono-nutrition data (meals number/day, night fasting, night eating, etc.), collected at the same time. Additional nutritional data (fermented and antioxidant food intake) were also collected using an ad hoc questionnaire. Waist circumference, weight, BMI and body composition data were collected using the OMRON BF511 medical device, and skeletal muscle mass index (SMMI) was calculated using the formula SMMI = [weight (kg) × (MM% (BIA)/100)]/height (m^2^) [31]. All these data were collected at baseline and the 3-, 6- and 12-month follow-up visits.

For the total score of the 17-items er-MEDAS [28], each of the 17 items was scored as 1 point if adhered to and 0 points otherwise. Therefore, a score ranging from 0 to 17 points was developed, with 0 meaning no adherence and 17 meaning maximum adherence. Subsequently, the score was categorized into approximate tertiles: low (≤7), medium (8–10) and high (11–17) [32]. The adherence to each specific item was also studied.

Furthermore, with the semiquantitative sFFQ, the weekly/monthly frequency of consumption of different food and food groups was collected, converted to consumed amount/day through portion intake calculations and studied following Rodríguez et al. (2008) [29].

To complete information, two 24 HR questionnaires were collected by a registered dietitian on two alternative days for each follow-up visit. Caloric energy, macronutrient and key micronutrient intake data were also estimated using the ODIMET program (www.odimet.es), a freeware computer program to calculate the estimated intake of macro and micronutrients [33].

The ODIMET findings were compared with the dietary reference values (DRVs) for nutrient intake established by the European Food Safety Authority (EFSA) [34]. For this, we used the DRV Finder, an interactive tool that provides quick and easy access to EFSA’s dietary reference values (DRVs) [35].

### 2.5. Statistical Analysis

The sampling size was calculated, with GRANMO version 8, assuming an alpha risk of 0.05 and a statistical power greater than 0.9 in a bilateral contrast. Thus, 68 subjects were required in each of the two groups to detect a difference equal to or greater than 1 unit in the main outcome er-MEDAS score. The common standard deviation was assumed to be 1.5. A loss to follow-up rate of 30% was estimated.

A descriptive analysis of the baseline sample was performed for the INT and CTR groups, using frequency and percentage for categorical variables and mean and standard deviation for continuous variables. To detect statistically significant differences between the two groups at baseline, a Z-test of differences in proportions and the non-parametric Mann-Whitney U test were performed, depending on the type of variables.

The er-MEDAS was described by mean, standard deviation and median according to the group to which it belonged, at baseline, 3, 6 and 12 months, respectively. The non-parametric Mann-Whitney U test was used to detect statistically significant differences between groups, while the Friedman and Wilcoxon tests were employed to assess differences within each group throughout the follow-up. 

Furthermore, adherence levels and adherence to each item were also studied at baseline, 3, 6 and 12 months. To detect statistically significant differences between the two groups, a Z-test of differences in proportions was conducted.

To detect statistically significant differences in the different er-MEDAS items between groups, the Z-test for differences in proportions and the McNemar’s test within each group were performed.

For anthropometric and chrono-nutritional variables, food (sFFQ) and nutrients (24 HR), a descriptive analysis of the baseline sample was performed using frequency and percentage for categorical variables and median and interquartile range by groups and at baseline, 3, 6 and 12 months. To detect statistically significant differences between groups, a Z-test of differences in proportions and the non-parametric Mann-Whitney U test were performed depending on the type of variables. To detect differences within each group throughout the follow-up, McNemar’s or the non-parametric Wilcoxon test was performed.

The effect of the intervention, time and group interaction on er-MEDAS was quantified with a linear mixed-effects models [36]. Potential associations of age, years since fibromyalgia diagnosis, education level and employment status were also studied. The analyses were performed with the IBM SPSS.20 statistical package and R version 4.2.2. Statistical significance was set at *p* < 0.05. The analyses were carried out with complete cases.

## 3. Results

### 3.1. The Study Sample

**Figure 2:**
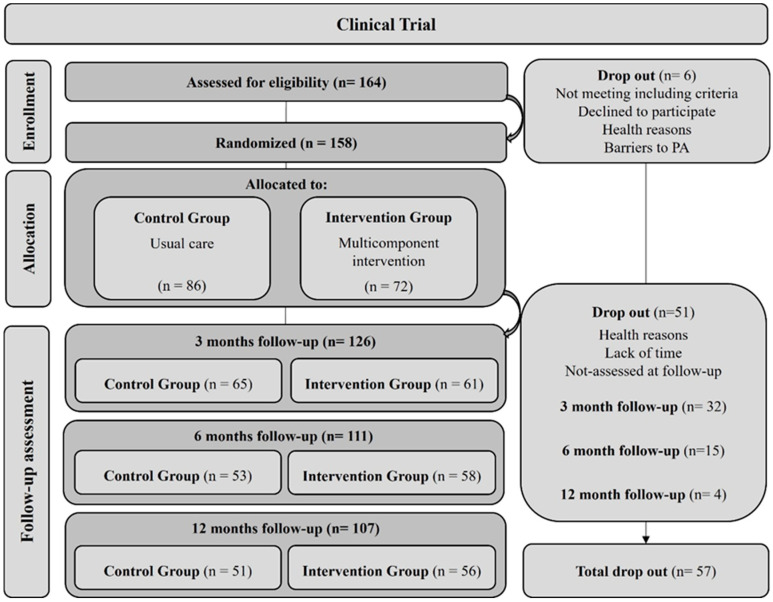
Sample flowchart of the study.

A total of 164 participants were recruited for the study. Of those, 158 participated in the study, and 72 were randomized and allocated to the INT group with 86 allocated to the CTR group at baseline. Ten intervention group sessions comprising 6–8 participants were held between October 2021 and May 2023. Follow-up visits ended in May 2024. At the 3-month follow-up, 61 and 65 participants were assessed in the INT and CTR groups, respectively; at 6 months post-intervention, 58 INT and 53 CTR were assessed; and at 12 months post-intervention, 56 INT and 51 CTR were assessed. Total loss to follow-up was 31.4% (Figure 2).

Table 1 presents relevant sociodemographic and socioeconomic characteristics of the study sample population. In short, study participants were mainly women 94.3% with FM, with a mean age of 50.5 (range 21–64, with most in the 40–60 range), and 6 years (mean) since FM diagnosis. A total of 58.2% were also diagnosed with CFS. Most of them had completed secondary education (39.2%), and 57% were not working at the moment of the study. A total of 39% percent were obese according to BMI, 29.1% were overweight, 1.9% were underweight and 49.4% had low muscle mass, with <6.7 SMMI index [31]. They were polypharmacy patients taking a mean of five prescribed medications. A significant number of participants (61.4%) were taking food supplements such as vitamin D, vitamin B12, iron, probiotics and melatonin. No significative differences were observed between the CTR and INT groups at the pre-intervention visit regarding the variables studied.

### 3.2. Mediterranean Diet Adherence Outcomes

**Figure 3:**
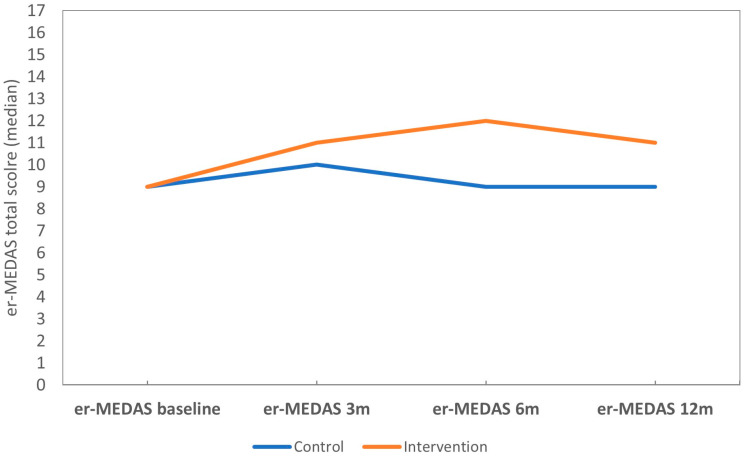
Total er-MEDAS score evolution in the study groups.

The INT group showed greater adherence to the 17-item er-MEDAS in the INT group after intervention, and differences observed between the groups at 3, 6 and 12 months were significant (*p* < 0.001, *p* < 0.001, *p* = 0.002, respectively) (Table 2) (Figure 3). Along the same lines, both groups improved er-MEDAS adherence initially. However, the INT group significantly improved adherence to the low-energy Mediterranean diet er-MEDAS at 3 months post-intervention (baseline median: 9 (IQR 4) vs. 3 months median: 11 (IQR 3), *p* < 0.001) (Table 3), and significant improvement was maintained at 6 and 12 months post-intervention (*p* < 0.001, *p* < 0.001, respectively). The results of the mixed linear model showed that the 17-item er-MEDAS adherence score depended on group-time interaction, being positive and significant for the INT group at 3 and 6 months and almost significant at 12 months (Table 4). Furthermore, age and education level also had an effect on er-MEDAS adherence, as scores were positive and significant for participants with higher levels of education.

More participants significantly improved adherence to er-MEDAS in the INT group at all post-intervention follow-ups: 73.8% at 3 months, 70.7% at 6 months, and 66.1% at 12 months post-intervention. By comparison, adherence in the CTR group was 50%, 48.1% and 46% at 3, 6 and 12 months post-intervention, respectively.

**Figure 4:**
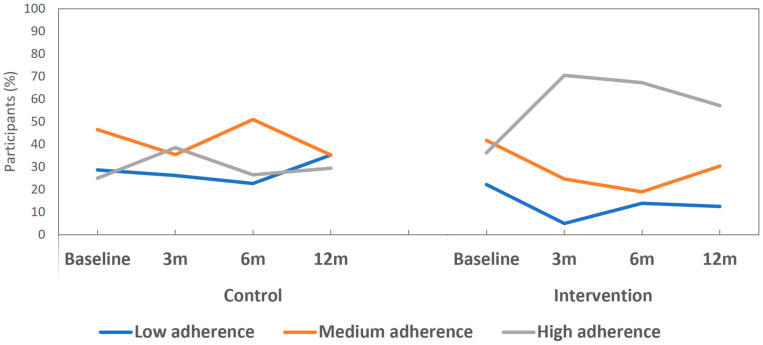
Levels of er-MEDAS adherence evolution in the study groups.

Levels of adherence to the 17-item er-MEDAS (low, moderate and high adherence) were also studied in both groups at the follow-up visits. Improvement was significantly higher in the INT group compared to the CTR group at all post-intervention follow-ups (*p* < 0.001, *p* = 0.002, *p* = 0.026, at 3, 6 and 12 months, respectively). We also observed how the rate of individuals with “high adherence” significantly increased in the INT group from 36.1% at baseline to 70.5% at 3 months post-intervention (*p* < 0.001) (Table 2). Meanwhile, the rate of participants with “low adherence” significantly decreased from 22.2% to 4.9% (*p* < 0.001). This pattern was maintained at 6 months post-intervention, with 67.2% with “high adherence” (*p* < 0.001) at 6 months and 57.1% (*p* = 0.004) at 12 months post-intervention. In the CTR group, “high adherence” improved slightly but not significantly at 3 months post-intervention and then decreased again over the medium to long term (Figure 4).

Differences between groups in adherence to each of the 17 items of the er-MEDAS were already observed at 3 months post-intervention (Table 2). At that time, the INT group participants had higher adherence to the consumption of fruits (47.5% vs. 26.2%; *p* = 0.013) and nuts (65.6% INT vs. 35.4 CTR; *p* = 0.01) and less to sweets and pastries than the CTR group participants. However, the main differences between groups were observed at 6 months post-intervention, with significantly higher consumption of vegetables, legumes, fish and nuts and lower consumption of red and processed meat, butter and cream, sugar-sweetened beverages and sweets and pastries in the INT group (Table 2). Consumption of legumes remained significantly higher at 12 months (58.1% vs. 38.4%; *p* = 0.033). Regarding intragroup changes and evolution, the INT group increased intake of fruit (*p* = 0.001), legumes (*p* = 0.031), fish (*p* = 0.019), nuts (*p* = 0.013) and white meat (*p* = 0.039) and reduced sweets and pastries compared to baseline (*p* = 0.039). Adherence to the consumption of fruit, fish and legumes was maintained in the medium and long term (6 and/or 12 months). Furthermore, increased intake of whole grains (*p* = 0.036) and reduced intake of red meat (*p* = 0.001) and butter (*p* = 0.06) were achieved at 6- and 12-month follow-ups. Meanwhile, the CTR group reduced legumes and fish intake at 6 months post-intervention and increased consumption of sugar-sweetened beverages (Table 3).

### 3.3. Food Intake Frequency and Nutritional Quality

Results estimated from the semiquantitative sFFQ showed a significant statistical difference between the study groups. At the 3-month follow-up, the INT group had higher consumption of yogurt (*p* = 0.018) (Table 5), salad (*p* = 0.001), vegetables (*p* = 0.007), legumes (*p* < 0.001), white meat (*p* = 0.006), blue fish (*p* = 0.002), shellfish (*p* = 0.002), soft cheese (*p* = 0.012), fruits (*p* = 0.048) and nuts (*p* = 0.02) and significantly lower consumption of candies (*p* = 0.032). At the 6-month follow-up, the INT group had significantly higher consumption of potatoes (*p* = 0.020) and white fish (*p* = 0.022). The consumption of salad (*p* = 0.037), white meat (*p* = 0.042), blue fish (*p* = 0.001) and nuts (*p* = 0.011) was maintained in the medium and long term, especially for legumes (*p* = 0.007 at 12 months) and fruits (*p* = 0.007 at 12 months), as well as the reduced intake of chips (*p* = 0.018) and candies (*p* = 0.002) (Table 5). Furthermore, when observing intragroup evolution, the INT group also significantly reduced their intake of muffins (*p* = 0.027) and croissants (*p* = 0.020) and increased egg consumption (*p* < 0.001). Few changes were observed in the CTR group; however, they improved blue fish intake.

The ad hoc questionnaire showed significantly greater consumption of fermented foods (yogurt, kefir, etc.) in the INT group at all follow-up visits (42.6% vs. 7.7%, *p* < 0.001 at 3 months; 41% vs. 13.2%, *p* = 0.001 at 6 months; 29.5% vs. 13.7%, *p* = 0.025 at 12 months) (Table 6). Furthermore, the INT group also significantly improved the intake of fruit rich in antioxidants (berries, grapes, etc.) at all follow-up visits (*p* = 0.001 at 3 months, *p* = 0.002 at 6 months, *p* = 0.021 at 12 months), as well as food rich in omega-3 (fish, seeds, nuts, etc.) at 3 months (*p* = 0.04) and 12 months post-intervention (*p* = 0.013). The intake of fruit rich in antioxidants and food rich in omega-3 was significantly higher in the INT group than the CTR group at 3 (*p* < 0.001) and 6 months post-intervention (*p* = 0.019) (Table 6).

### 3.4. Dietary Intake Patterns

The INT group’s estimated energy intake using the 24 HR questionnaire increased at 3 months post-intervention from a median of 1166.12 Kcal/day (IQR 464.63) at baseline to a median of 1237.03 Kcal/day (IQR 499.58) (*p* = 0.029) (Table 7). No significant differences between groups regarding energy intake were observed. Macronutrient intake over total energy was similar in both groups: carbohydrates (CH) (42–44% CTR; 41–43% INT), fat (37.5–38.22% CTR; 38.92–40.3% INT), protein (18.37–19.49% CTR; 18.52–19.90% INT). As for daily protein consumption (gr/day), intake increased significantly in the INT group at 3, 6 and 12 months post-intervention compared to baseline (*p* = 0.011, *p* = 0.048, *p* = 0.040, respectively). Differences between groups for protein consumption were close to being significant at 12 months post-intervention (*p* = 0.056).

There was an increase (non-significant) in sugar intake in the CTR group at 6 and 12 months post-intervention, reaching a median of 39.62 g/day (IQR 29.65) vs. 29.45 g/day (IQR 32.01) at baseline. Likewise, there was a decrease (non-significant) in sugar intake in the INT group, going from a median of 31.95 g/day (IQR 28.50) at baseline to 29.19 g/day (IQR 21.07) at 12 months. Differences at 12 months were close to significant (*p* = 0.088). Fiber intake was low, with a median between 9.02–12.54 g/day. Fiber intake increased significantly in the INT group at 3 months post-intervention from 9.02 g/day (IQR 8.36) at baseline to 12.54 g/day (IQR 11.70) at 3 months (*p* = 0.006). Regarding key micronutrients, the INT group increased iron intake and achieved significantly greater intake at 12-months post-intervention than the CTR group (median 6.78 (IQR 5.02) vs. 4.94 (IQR 3.25; *p* = 0.014). 

Regarding chrono-nutrition, the rate of participants with night eating (between 12 am and 6 am) decreased in the INT group (19.4% at baseline vs. 13.1–8.2% at post-intervention follow-up visits), showing statistically significant differences between groups at 12 months post-intervention (8.2% INT participants vs. 25.5% CTR participants, *p* = 0.022). There was an increase in the CTR group in the number of night eaters from 17.9% at baseline to 25.5% at the 12-month follow-up. The number of meals per day was four to five in both groups at all follow-up visits with no significant differences. Night fasting (time between dinner and breakfast) was similar in both groups at all follow-up visits, with a median of 10–11 h/night. At 12 months post-intervention, night fasting was significantly greater in the INT group (median of 11 h/night and IQR 2; *p* = 0.016).

### 3.5. Anthropometry and Body Composition

No significant changes were observed between groups for BMI or waist circumference. SMMI significantly increased in the INT group at 6 and 12 months post-intervention (*p* < 0.001, in both cases) (Table 7), while it decreased in the CTR group (*p* = 0.001 at 6 months and *p* < 0.001 at 12 months). When regarding SMMI categories, “low SMMI” decreases for the INT group from 47.2% at baseline to 45.9% at 3 months, 46.7% at 6 months and 38.6% at 12 months, while it increased for the CTR group from 51.2% at baseline to 52.2% at 3 months, 53.7% at 6 months and 55.8% at 12 months. Differences between groups at 12 months post-intervention were close to being significant (38.6% INT group vs. 55.8% CTR group, *p* = 0.073).

## 4. Discussion

FM is mostly diagnosed in women, and, therefore, the majority of participants in the study were also women, aged mainly between 40–60, with a mean age of 50 years old. More than half of the participants were diagnosed with CFS. This syndrome is underdiagnosed [37], particularly in FM patients, and those who have not been diagnosed with CFS often complain of chronic fatigue. Most of them had completed secondary education and were not working at the moment of the study, and a high proportion were obese or overweight. Half of them had low muscle mass index. Most of the participants were polypharmacy patients; however, no improvements in symptoms, pain, fatigue or insomnia were perceived. Adherence to the Mediterranean diet was moderate at baseline, and the lifestyle change approach, based on expanding their nutritional knowledge, was new for most of them. The population’s knowledge of nutrition principles is insufficient and must be urgently addressed.

### 4.1. Mediterranean Diet Adherence

Adherence to the Mediterranean diet was studied using the energy-restricted 17-item Mediterranean Diet Adherence Screener er-MEDAS. This tool was developed by the PREDIMED-Plus project to evaluate interventions aimed at promoting weight loss for cardiovascular disease prevention [28]. However, it is a useful tool for several objectives. In our study, it was profitable because it assesses adherence to a Mediterranean diet that prioritizes the consumption of whole grains over white bread and refined cereals. Increasing micronutrients and fiber consumption, reducing post-prandial glucose spikes and achieving higher nutritional density were all part of our objectives. Consumption of the questionnaire items promotes a healthy Mediterranean-based diet pattern, rich in anti-inflammatory foods (fish, nuts, etc.), antioxidants, and fiber (vegetable, fruits, legumes, etc.) and with reduced proinflammatory and unhealthy foods (red and processed meat, saturated fats, refined sugars, etc.). Such a pattern has been identified as a suitable treatment for FM and CFS [21], and it is aligned with our geographical situation and eating habits. The INT group showed significant improvement in er-MEDAS adherence, increasing consumption of vegetables, legumes, fish, nuts and whole grains and lowering consumption of red and processed meat, butter and cream, sugar-sweetened beverages and sweets and pastries. Legume intake was specially improved and maintained in the long term. However, olive oil consumption did not increase as expected. This result needs to be improved, since olive oil has an additional beneficial impact on health outcomes [6,7,9,10].

The results of the mixed linear model showed that the 17-item er-MEDAS adherence score depended on group-time interaction, being positive and significant for the INT group at 3 and 6 months and almost significant at 12 months. Age and education level were relevant in the effectiveness of the intervention on er-MEDAS adherence, as it was higher for participants with a higher education and older age.

The effectiveness of the intervention on improving adherence to the Mediterranean diet was relevant, considering that the nutrition sessions totaled just 6 h, and they were accompanied by home material, WhatsApp discussions and quick reminders at follow-up visits. This is a relevant achievement considering the difficulty of originating lifestyle changes in the general population, even when transmitting adequate education and knowledge. Thus, it is important for this population profile considering that their quality of life is affected by pain, fatigue and insomnia and sometimes digestive problems, anxiety and depression. However, associated health outcome improvements must be further studied. 

Adherence improvement in the INT group is important, taking into account various relevant associated health benefits: cardiovascular, metabolic syndrome, mental health, digestive health and healthy weigh maintenance, among others [1,2,3,4,5,6,7,8,9,10]. Previous studies demonstrated that a Mediterranean diet enriched with walnuts had beneficial effects on fatigue, anxiety, depression and eating disorders in women with FM [38]. Moreover, near daily intake of fruit and vegetables and moderate intake of fish were associated with better psychosocial outcomes in women with FM [39]. The impact of our intervention on health outcomes is currently under study.

### 4.2. Food Intake Frequency and Nutritional Quality

Results estimated from the semiquantitative sFFQ were in line with those observed in the er-MEDAS, showing a significant increase in consumption of salad, vegetables, legumes, white meat, blue fish, shellfish, soft cheese, fruits and nuts and significantly reduced consumption of red and processed meat, sweets and chips. Consumption remained increased in the medium and long term for legumes, blue fish and nuts and reduced for chips and sweets. Thus, intake of healthy and nutritious foods improved, while unhealthy food intake and the consumption of empty calories was reduced. Furthermore, the SYNCHRONIZE + intervention participants improved their intake of fermented foods and foods rich in antioxidants. This is relevant considering that fermented food intake is associated with a healthy gut microbiota [40]. On the other hand, antioxidant intake is known to balance oxidative stress, which is usually altered in these patients [41]. Thus, the intervention increased global nutritional quality and gut microbiota health.

### 4.3. Dietary Intake Patterns

Energy intake increased in the INT group, with a maximum median value of 1237.03 Kcal/day. One of the objectives of the intervention was to increase caloric intake and nutritional density, along with physical activity. Participants complained of fatigue, and at baseline, energy intake was sometimes insufficient, based on empty calories with low nutritional value (sweets, pastries, sugar-sweetened beverages, etc.). The EFSA reference values for nutrient intake for women aged 40–59 from the healthy population [34] indicate that the average energy requirement for low physical activity (PAL 1.4) is 1783–1798 Kcal/day. However, a considerable proportion of study participants were obese or overweight. Unhealthy energy intake, sedentarism, insomnia, medication and metabolic factors may all play a role. Insufficient caloric intake or altered intake patterns must be further identified and the potential causes studied (ex. metabolic disorders, anxiety, depression, eating disorders, digestive issues, body perception, restriction diets, binge eating disorder, night eating syndrome, etc.).

The reference EFSA range for total macronutrient intake for healthy women aged 40–59 is 45–60% for CH, while adequate fiber intake is 25 g/day. The reference range for total fats intake is 20–35%. The protein intake reference for the healthy population is 0.83 g protein/kg weight/day. Study participants’ macronutrient intake over total energy was similar in both groups. Values were for 43–44% CH approximately 37–40% fat and 18–20% protein. Thus, CH consumption was lower, but close to the EFSA range, while fats were higher. However, the consumption of blue fish, nuts and healthy fat was promoted. The INT group increased protein intake. Adequate consumption of protein is relevant in FM, since it helps increase SMMI, along with physical activity. Protein intake is also beneficial in promoting adequate levels of neurotransmitters and mental health. Thus, these findings are promising if we consider that a higher percentage of protein intake is associated with a higher pain threshold in FM [42]. In addition, the role of essential amino acids such as tryptophan and its metabolites, such as serotonin and kynurenine, are being studied in FM for their connection with inflammation, the immune system, neurological conditions, pain and fatigue [43,44]. Furthermore, one of the main results of the project is the increased consumption of legumes and nuts, which represent healthier and sustainable plant-based protein. However, even so, fiber intake was low in general, with a median of 9.02–12.54 g/day, when the EFSA recommended intake is 25 g/day. This must be improved by educating about the importance of increasing whole grains and vegetables. Adequate fiber intake has been associated with interesting health outcomes and gut microbiota health [45].

Regarding micronutrients, the EFSA reference intake for healthy women aged 40–59 of iron is 11–16 mg/day; vitamin B12 is 4 µg/day; vitamin D is 15 mg/day; vitamin E is 11 mg/day; vitamin C is 80–95 mg/day and calcium is 750–905 mg/day. We also studied iron intake, since it is a key nutrient. Study participants had low iron intake of around 5.5 mg/day, which increased in the INT group to 6–7 mg/day. However, this was still insufficient intake given the EFSA recommendation of 11–16 mg/day. Future intervention must reinforce this point. However, not only intake, but also gut microbiota health and other factors affecting micronutrient intestinal absorption must be taken into account.

Micronutrient deficiencies and low plasmatic levels of certain vitamins and minerals including iron (low levels of plasma ferritin), vitamin B12 and vitamin D have been observed regularly in FM patients [46,47,48]. Furthermore, Bjorklund et al. (2018) [49] concluded that when optimal levels of nutrients are achieved, pain levels usually decrease. A primary care pilot study conducted in Catalonia associated FM diagnosis with low levels of plasmatic vitamin D and/or ferritin, as well as a thyroid autoimmune dysfunction (Valls-Llobet, personal communication, unpublished results). In this line, several studies have pointed out that increasing antioxidant intake, improving digestive symptoms and preventing or rebalancing deficiencies of certain micronutrients can improve FM symptoms [47,50,51,52,53]. Thus, further research is needed on the importance of regularly monitoring key micronutrient levels (ferritin, vitamin D and vitamin B12, among others) in persons with FM, since improving dietary intake and supplementing when required under medical advice seems to be of therapeutical relevance (Carrasco-Querol et al., 2024) [21]. Micronutrient deficiencies need to be regularly monitored in FM, especially ferritin, vitamin D and vitamin B12.

As for chronobiology, one major achievement was reducing the number of INT group participants with night eating, with significant differences between groups at 12 months post-intervention. Night eating is a clear chrono-disruptor that alternates circadian rhythms with metabolic consequences [54]. Night fasting was around 11 h, which is close to the objective of 12–13 h. Night fasting of at least 12 h has shown health benefits [54,55,56].

### 4.4. Anthropometry and Body Composition

No significative changes were observed between groups for BMI and waist circumference. However, SMMI increased in the INT group at 6 and 12 months post-intervention, while it decreased in the CTR group. Thus, “low SMMI” decreased in the INT group, while it increased in the CTR group. Changes in muscle mass are usually associated with an adequate protein intake combined with physical activity, also included in the intervention. Differences between groups at 12 months post-intervention were close to being significant. Higher muscle mass is associated with better psychological health, less widespread pain and a lower impact of FM symptoms [57,58]. Furthermore, a recent study identified a significant reduction in muscle function (dynapenia) in FM patients without any significant loss of muscle mass [59]. Dynapenia needs to be further studied and approached in FM. Our patients have also shown an increase in body strength in the INT group [60].

### 4.5. Study Limitations

Most of the data collected were self-reported and non-objective and thus could be over- or underestimated by the participants when reported. Specifically, sFFQ might overestimate the intake of certain food groups. Furthermore, external factors not controlled by the study (TV publicity, changes in food prices, friends and family influences, etc.) could be affecting our results, since it was conducted in a real-life primary care clinical setting. The study was performed with participants with <10 years since FM diagnosis, and results cannot be generalized for other timeframes. 

### 4.6. SYNCHRONIZE + Intervention

The SYNCHRONIZE + is a short, low-cost, multidisciplinary intervention (nutrition, chronobiology and physical activity education) with 6 h of nutritional sessions, effective in improving adherence to the Mediterranean diet, increasing the consumption of fruit, legumes, nuts, fruits, whole grains and blue fish and reducing red and processed meat, sweets, pastries and butter in people with FM, with or without CFS, through primary care. To ensure its effectiveness and achieve the established goals, it is crucial to have a registered dietitian/nutritionist developing nutritional sessions and follow-up visits. The dietitian/nutritionist plays a key role in primary care in the prevention and management of chronic diseases. SYNCHRONIZE + can be implemented in primary care as a complementary intervention [61,62] or a brief independent one for people with limited availability due to work or family reasons. Furthermore, its effectiveness has been demonstrated in improving adherence to the Mediterranean diet, nutritional intake patterns (this study), and physical activity levels, body strength and cardiorespiratory capacity [60]. SYNCHRONIZE + can be easily used and adapted to other chronic diseases and objectives. Future studies on the management of FM and CFS should include an individualized approach to complement the group approach.

## 5. Conclusions

SYNCHRONIZE + is a brief low-cost multidisciplinary intervention with attractive potential for improving nutritional habits and lifestyle in FM and CFS and, potentially, for chronic disease management and promoting health. Further evaluation of the effectiveness of the SYNCHRONIZE + intervention on improving quality of life and symptoms in people with FM and CFS is required.

## Figures and Tables

**Table 1:** Baseline characteristics of overall sample and by treatment condition.

	Overall (*n* = 158)	CTR Group (*n* = 86)	INT Group (*n* = 72)	*p*
***Socio-demographics***				
**Age** (years), mean (SD) **Female**, n (%) **Education level**, n (%) Basic studies Secondary studies Higher university studies **Employment status**, n (%) Employed Unemployed	50.5 (7.9) 149 (94.3) 26 (16.5) 62 (39.2) 16 (10.1) 66 (41.8) 90 (57)	50.6 (7.7) 80 (93) 15 (17.4) 32 (37.2) 8 (9.3) 36 (41.9) 48 (55.8)	50.4 (8.15) 69 (95.8) 11 (15.3) 30 (41.7) 8 (11.1) 30 (41.7) 42 (58.3)	0.849 0.307 0.715 0.568 0.707 0.980 0.750
***Clinical data***				
**Medications** (num), mean (SD) **Years with FM diagnosis**, mean (SD) **Years with FM diagnosis**, n (%) Less than 1 year Between 1 and 5 years More than 5 years **Chronic fatigue diagnosis**, n (%)	5 (3) 6.8 (4.9) 0 (0) 73 (46.2) 78 (49.4) 92 (58.2)	5 (3) 6.8 (4.6) 0 (0) 42 (48.8) 38 (44.2) 53 (61.6)	5 (4) 6.8 (5.25) (0) 31 (43.1) 40 (55.6) 39 (54.2)	0.719 0.786 --- 0.468 0.155 0.344
***Anthropometry data***				
**Waist circumference** (cm), mean (SD) **SMMI**, mean (SD) **SMMI categories**, n (%) Low muscle mass Normal muscle mass **BMI**, mean (SD) **BMI categories**, n (%) Underweight Normal Overweight Obese	97.7 (15.1) 6.9 (1.1) 78 (49.4) 80 (50.6) 28.8 (6.3) 3 (1.9) 46 (29.1) 47 (29.7) 62 (39.2)	97.8 (15.4) 7 (1.2) 44 (51.2) 42 (48.8) 29.2 (6.5) 0 (0) 24 (27.9) 27 (31.4) 35 (40.7)	97.5 (14.9) 6.9 (1.1) 34 (47.2) 38 (52.8) 28.2 (6) 3 (4.2) 22 (30.6) 20 (27.8) 27 (37.5)	0.918 0.883 0.622 0.622 0.461 0.056 0.715 0.620 0.682
***Diet***				
**Type of diet**, n (%) Omnivorous Ovo-lacto vegetarian **Mediterranean Diet Adherence** *, mean (SD) **Type of food**, n (%) Fermented food Polyphenols rich fruits ^1^ Other polyphenols foods ^2^ Omega-3 rich food ^3^ Supplement (Vit D, iron, Vit B12, others)	153 (96.8) 3 (1.9) 9.3 (2.6) 26 (16.5) 45 (28.5) 63 (39.9) 76 (48.1) 97 (61.4)	82 (95.3) 2 (2.3) 9 (2.7) 11 (13.1) 28 (33.3) 31 (36.9) 44 (52.4) 51 (59.3)	71 (98.6) 1 (1.4) 9.5 (2.5) 15 (20.8) 17 (23.6) 32 (44.4) 32 (44.4) 46 (63.9)	0.243 0.667 0.223 0.196 0.181 0.339 0.323 0.555
***Chronobiology***				
**Meals/day**, n (%) **Night eating**, n (%) **Night fasting time**, mean (SD)	4.5 (1) 29 (18.4) 10.7 (2.4)	4.3 (1) 15 (17.4) 10.6 (2.3)	4.7 (1) 14 (19.4) 10.8 (2.6)	0.891 0.746 0.344

CTR group: control group; INT group: intervention group; SD: standard deviation; FM: fibromyalgia; SMMI: skeletal muscle mass index; BMI: body mass index. * er-MEDAS total score. ^1^ e.g., red berries, grapes, blueberries; ^2^ e.g., green tea, dark chocolate, pure cacao; ^3^ e.g., nuts and seeds. *p*: Mann Whitney U-test for continuous data and chi-square test for categorical data.

**Table 2:** Effects of the intervention by group and time for Mediterranean Adherence Diet (er-MEDAS 17-items).

	CTR Group				INT Group				CTR vs. INT (*p*-Values)			
	0 Month	3 Months	6 Months	12 Months	0 Month	3 Months	6 Months	12 Months	0 m	3 m	6 m	**12 m**
**er-MEDAS adherence**, n (%) **Low (0–7)**	24 (28.6)	17 (26.2)	12 (22.6)	18 (35.3)	16 22.2)	3 (4.9)	8 (13.8)	7 (12.5)	0.365	**0.001**	0.226	0.005
**Moderate (8–10)**	39 (46.4)	23 (35.4)	27 (50.9)	18 (35.3)	30 (41.7)	15 (24.6)	11 (19)	17 (30.4)	0.551	0.187	**<0.001**	0.587
**High (11–17)**	21 (25)	25 (38.5)	14 (26.4)	15 (29.4)	26 (36.1)	43 (70.5)	39 (67.2)	32 (57.1)	0.132	**<0.001**	**<0.001**	**0.004**
**er-MEDAS 17-items**, n (%) 1. Use extra-virgin olive oil 2. Vegetables, ≥2 portions/day 3. Fruits, ≥3 portions/day 4. Red meat, ≤1 serving/week 5. Butter, <1 serving/week 6. Sweet beverages, <1/week 7. Legumes, ≥3 servings/week 8. Fish, ≥3 servings/week 9. Sweets and pastries, <3/week 10. Nuts, ≥3 servings/week 11. Preference white meat 12. Sofrito, ≥2 times/week 13. Adding sugar to beverages 14. White bread, <1 serving/day 15. Whole grains, ≥5 times/week 16. Refined cereals, <3 servings/week 17. Wine, 2–3 or 1–2 glasses/day *	78 (90.7) 46 (53.5) 24 (27.9) 33 (38.4) 66 (76.7) 57 (66.3) 25 (29.1) 24 (27.9) 56 (65.1) 35 (40.7) 70 (81.4) 53 (61.6) 37 (43) 58 (67.4) 23 (26.7) 52 (60.5) 11 (12.8)	63 (96.9) 39 (60) 17 (26.2) 29 (44.6) 54 (83.1) 46 (70.8) 19 (29.2) 24 (36.9) 48 (73.8) 23 (35.4) 57 (87.7) 40 (61.5) 28 (43.1) 46 (70.8) 19 (29.2) 44 (67.7) 8 (12.3)	50 (94.3) 28 (52.8) 14 (26.4) 22 (41.5) 43 (81.1) 35 (66) 11 (20.8) 10 (18.9) 36 (67.9) 23 (43.4) 48 (90.6) 37 (69.8) 25 (47.2) 37 (69.8) 20 (37.7) 36 (67.9) 7 (13.2)	46 (90.2) 32 (62.7) 16 (31.4) 23 (45.1) 41 (80.4) 36 (70.6) 16 (31.4) 0 (0) 38 (74.5) 25 (49) 46 (90.2) 36 (70.6) 27 (52.9) 35 (68.6) 20 (39.2) 31 (60.8) 7 (13.7)	66 (91.7) 40 (55.6) 16 (22.2) 29 (40.3) 58 (80.6) 53 (73.6) 20 (27.8) 22 (30.6) 55 (76.4) 34 (47.2) 59 (81.9) 54 (75) 26 (36.1) 52 (72.2) 20 (27.8) 44 (61.1) 17 (23.6)	60 (98.4) 42 (68.9) 29 (47.5) 33 (54.1) 54 (88.5) 51 (83.6) 25 (41) 30 (49.2) 54 (88.5) 40 (65.6) 58 (95.1) 47 (77) 18 (29.5) 50 (82) 27 (44.3) 40 (65.6) 9 (14.8)	56 (96.6) 43 (74.1) 23 (39.7) 36 (62.1) 56 (96.6) 50 (86.2) 26 (44.8) 30 (51.7) 49 (84.5) 38 (65.5) 53 (91.4) 43 (74.1) 17 (29.3) 40 (69) 19 (32.8) 38 (65.5) 12 (20.7)	56 (100) 36 (64.3) 25 (44.6) 29 (51.8) 53 (94.6) 48 (85.7) 29 (51.8) 0 (0) 48 (85.7) 33 (58.9) 49 (87.5) 35 (62.5) 18 (32.1) 46 (82.1) 27 (48.2) 36 (64.3) 17 (30.4)	0.831 0.795 0.413 0.807 0.562 0.318 0.858 0.715 0.123 0.410 0.929 0.073 0.377 0.515 0.884 0.934 0.076	0.597 0.300 **0.013** 0.287 0.382 0.087 0.167 0.165 **0.036** **0.001** 0.142 0.060 0.114 0.140 0.080 0.801 0.688	0.575 **0.020** 0.139 **0.030** **0.009** **0.012** **0.007** **<0.001** **0.040** **0.019** 0.881 0.612 0.053 0.923 0.583 0.788 0.296	**0.016** 0.869 0.158 0.489 **0.024** 0.057 **0.033** --- 0.145 0.304 0.659 0.376 **0.030** 0.104 0.349 0.708 0.039
**er-MEDAS total score**, median (IQR)	9 (3.5)	10 (4)	9 (3)	9 (5)	9 (4)	11 (3)	12 (4)	11 (3.5)	0.230	**<0.001**	**<0.001**	**0.002**
*Friedman test*	CTR group				INT group							
*p*-value between all visits	0.214				**<0.001**							
Chi square	4.480				39.190							

NOTE: Each er-MEDAS item refers to compliance with food habits (Schröder et al., 2021) [28]. * Men and women, respectively. The number of participants varied across assessments (see Figure 1). Bold fonts stand for significant values.

**Table 3:** Intragroup change by time for Mediterranean Adherence Diet (er-MEDAS 17-items).

Mediterranean Adherence Diet er-MEDAS 17-Items	CONT Group			INT Group		
	0 vs. 3 m	**0 vs. 6 m**	**0 vs. 12 m**	**0 vs. 3 m**	**0 vs. 6 m**	**0 vs. 12 m**
1. Use extra-virgin olive oil 2. Vegetables, ≥2 portions/day 3. Fruits, ≥3 portions/day 4. Red meat, ≤1 serving/week 5. Butter, <1 serving/week 6. Sweet beverages, <1/week 7. Legumes, ≥3 servings/week 8. Fish, ≥3 servings/week 9. Sweets and pastries, <3/week 10. Nuts, ≥3 servings/week 11. Preference white meat 12. Sofrito, ≥2 times/week 13. Adding sugar to beverages 14. White bread, <1 serving/day 15. Whole grains, ≥5 times/week 16. Refined cereals, <3 servings/week 17. Wine, 2–3 or 1–2 glasses/day *	0.219 0.999 0.999 0.332 0.424 0.388 0.999 0.146 0.332 0.629 0.999 0.581 0.999 0.581 0.999 0.405 0.625	0.625 0.629 0.999 0.999 0.508 **<0.001** **0.001** **0.039** 0.999 0.999 0.687 0.629 0.687 0.388 0.096 0.523 0.999	0.999 0.508 0.999 0.302 0.999 0.205 0.581 --- 0.149 0.581 0.289 0.549 0.999 0.549 0.096 0.815 0.999	0.250 0.096 **0.001** 0.052 0.227 0.065 **0.031** **0.019** **0.039** **0.013** **0.039** 0.999 0.424 0.238 0.093 0.648 0.219	0.999 **0.006** **0.049** **0.001** **0.006** 0.999 0.265 **0.012** 0.332 0.064 0.227 0.999 0.388 0.999 0.999 0.791 0.999	NA 0.302 **0.007** **0.049** **0.021** 0.109 **<0.001** --- 0.267 0.096 0.344 0.267 0.447 0.210 **0.036** 0.999 0.388

* Men and women, respectively. Bold fonts stand for significant values.

**Table 4:** Estimated association between Mediterranean Diet Adherence (er-MEDAS 17-items) and intervention participation adjusted for socio-demographics and clinical data using GMM.

	ß/Estimate	*p* Value
**Intercept**	5.76	**<0.001**
***Socio-demographics*** **Age** **Education level** Basic studies Secondary studies Higher university studies **Employment status**	0.05 1 0.65 2.12 −0.13	**0.036** 0.229 **0.004** 0.745
***Clinical data*** **Years since FM diagnosis**	−0.04	0.327
***Group and time*** **Group** Control Intervention **Time** 0-month 3-month 6-month 12-month **Group-time interaction** Intervention: 3-month Intervention: 6-month Intervention: 12-month	1 0.48 1 0.45 0.20 0.41 1.43 1.38 0.82	0.258 0.109 0.519 0.179 **<0.001** **0.001** **0.055**
**SD intercept** **SD residual**	1.99 1.61	**<0.001**

GMM: generalized mixed model; FM: fibromyalgia.

**Table 5:** Effects of the intervention by group and time for short food frequency questionnaire (sFFQ).

SFFQ List of Foods	CTR Group, Median (IQR)				INT Group, Median (IQR)				CTR vs. INT (*p*-Values)			
Adherence to the Mediterranean Diet	0 Month	3 Months	6 Months	12 Months	0 Month	3 Months	6 Months	12 Months	0 m	3 m	6 m	12 m
1. Milk	220 (198)	220 (188.6)	220 (220)	220 (220)	220 (220)	220 (220)	220 (220)	188.6 (220)	0.176	0.676	0.848	0.828
2. Yogurt	53.6 (89.3)	53.6 (89.3)	53.6 (63.1)	71.4 (112.5)	53.6 (71.4)	71.4 (35.7)	71.4 (94.6)	53.6 (56)	0.736	**0.018**	0.317	0.816
3. Bonbons	0.7 (5.7)	1 (5.7)	1.3 (2.9)	1.3 (5.7)	2.7 (5.7)	1.3 (2.9)	0 (3.3)	0 (1.3)	0.541	0.819	0.291	**0.005**
4. Breakfast cereals	0 (0)	0 (0.6)	0 (2.3)	0 (0)	0 (1.2)	0 (0)	0 (0)	0 (0)	0.213	0.458	0.202	0.845
5. Butter cookies	0 (12.9)	0 (8.60)	0 (4.3)	0 (6)	0 (5)	0 (8.6)	0 (4)	0 (4)	0.274	0.754	0.677	0.601
6. Chocolate cookies	0 (2.3)	0 (2.3)	0 (2.3)	0 (0)	0 (2.3)	0 (0)	0 (0)	0 (0)	0.706	0.069	0.438	0.870
7. Muffins	0 (8.6)	0 (3)	0 (4)	0 (4)	1 (8.6)	1 (4.3)	0 (2)	0 (2)	0.371	0.114	0.962	0.621
8. Croissants	0 (6.4)	0 (6.4)	0 (6.4)	3 (6.4)	1.5 (6.4)	0 (6.4)	0 (3)	0 (6)	0.572	0.469	0.131	0.308
9. Salad	71.4 (57.1)	57.1 (71.4)	71.4 (42.9)	66.7 (57.1)	71.4 (57.1)	85.7 (28.6)	71.4 (42.9)	85.7 (42.9)	0.754	**0.001**	**0.037**	**0.077**
10. Vegetables	85.7 (85.7)	85.7 (81)	85.7 (57.2)	85.7 (85.7)	60 (57.2)	85.7 (28.6)	85.7 (57.2)	85.7 (57.2)	0.412	0.307	0.952	0.543
11. Vegetable garnish	42.9 (42.9)	34.3 (42.9)	42.9 (28.6)	40 (42.9)	42.9 (28.6)	42.9 (42.9)	42.9 (42.9)	42.9 (42.9)	0.763	**0.007**	0.232	0.101
12. Potatoes	42.9 (44.3)	64.3 (22.9)	42.9 (48.6)	42.9 (42.9)	42.9 (64.3)	64.3 (42.9)	64.3 (42.9)	42.9 (64.3)	0.323	0.057	**0.020**	0.455
13. Legumes	17.1 (8.6)	17.1 (8.6)	17.1 (17.1)	17.1 (17.1)	17.1 (8.6)	25.7 (8.6)	17.1 (8.6)	20 (8.6)	0.716	**<0.001**	**0.007**	**0.007**
14. Rice	10 (10)	10 (10)	10 (10)	10 (10)	20 (10)	20 (10)	10 (10)	18.7 (10)	0.423	0.692	0.196	0.147
15. Pasta	20 (10)	20 (20)	10 (13)	10 (10)	20 (20)	20 (20)	20 (20)	20 (10)	0.635	0.762	0.088	0.219
16. Soup	4.3 (8.6)	8.6 (10.9)	7.3 (8.6)	8.6 (8.6)	8.6 (9.7)	8.6 (8.6)	8.6 (8.6)	8.6 (8.6)	**0.008**	0.113	0.056	0.381
17. Eggs	23.6 (7.9)	23.6 (15.7)	23.6 (15.7)	23.6 (15.7)	23.6 (14.9)	39.3 (23.6)	31.4 (15.7)	31.4 (23.6)	**0.022**	**<0.001**	**0.002**	0.271
18. Chicken or turkey	64.3 (42.9)	64.3 (42.9)	64.3 (45.7)	64.3 (45.7)	64.3 (42.9)	85.7 (21.4)	85.7 (42.9)	64.3 (42.9)	0.644	**0.006**	**0.042**	0.362
19. Red meat	21.4 (32.9)	21.4 (37.9)	21.4 (32.9)	21.4 (37.9)	21.4 (27.9)	21.4 (37.9)	21.4 (22.9)	21.4 (21.4)	0.700	0.661	0.385	0.213
20. Burgers	14.3 (18.6)	14.3 (16.9)	14.3 (23.3)	14.3 (28.6)	14.3 (11)	14.3 (28.6)	14.3 (28.6)	14.3 (14.3)	0.130	0.070	0.730	**0.027**
21. White fish	21.4 (32.9)	42.9 (27.9)	41.4 (22.9)	21.4 (42.9)	42.9 (42.9)	42.9 (42.9)	42.9 (42.9)	42.9 (28.6)	0.228	0.110	**0.022**	0.066
22. Blue fish	21.4 (37.9)	21.4 (32.9)	35 (41.4)	36.4 (52.9)	21.4 (32.9)	42.9 (42.9)	57.9 (36.4)	51.4 (52.9)	0.159	**0.002**	**0.001**	0.195
23. Shellfish	7.1 (7.1)	3.3 (7.1)	1.7 (3.3)	1.7 (3.3)	7.1 (5.5)	7.1 (9.3)	1.7 (3.3)	1.7 (1.7)	0.635	**0.002**	0.481	0.338
24. Pizza	5.3 (8.8)	9.3 (11.4)	2.7 (10.7)	2.7 (11.4)	11.4 (8.8)	5.3 (11.4)	2.7 (11.4)	2.7 (22.9)	0.817	0.286	0.720	0.444
25. Bread	19.3 (39)	12.9 (40.3)	0 (3)	0 (0)	32.1 (32.1)	32.1 (32.1)	0 (0)	0 (10.5)	**0.037**	**0.031**	0.777	0.106
26. Processed meat	10.7 (10.7)	7.1 (7.4)	7.1 (10.7)	7.1 (14.3)	10.7 (7.2)	7.1 (10.7)	7.1 (10.7)	7.1 (14.3)	0.670	0.981	0.752	0.911
27. Fresh/soft cheese	7.1 (10.7)	7.1 (10.7)	7.1 (14.3)	7.1 (10.7)	7.1 (10.7)	10.7 (10.7)	10.7 (10.7)	7.1 (10.7)	0.588	**0.012**	0.180	0.271
28. Cured cheese	7.1 (10.7)	7.1 (9.9)	5.4 (9)	3.6 (10.7)	3.6 (10.7)	3.6 (10.7)	3.6 (10.7)	3.6 (10.7)	0.691	0.393	0.759	0.717
29. Citrus	42.9 (71.4)	28.6 (62.6)	28.6 (57.1)	42.9 (57.1)	42.9 (71.4)	42.9 (100)	42.9 (71.4)	28.6 (57.1)	0.274	0.166	0.249	0.587
30. Other fruits	42.9 (42.9)	57.1 (71.4)	57.1 (64.8)	57.1 (57.1)	71.4 (57.1)	71.4 (57.1)	71.4 (42.9)	71.4 (42.9)	**0.006**	**0.048**	**0.016**	**0.007**
31. Fruit in syrup	0 (0)	0 (0)	0 (0)	0 (0)	0 (0)	0 (0)	0 (0)	0 (0)	0.861	0.539	0.683	0.050
32. Natural juices	0 (28.6)	0 (24.3)	0 (0)	0 (13.3)	0 (13.3)	0 (28.6)	0 (6.7)	0 (40)	0.296	0.917	0.584	0.153
33. Commercial juices	0 (0)	0 (0)	0 (0)	0 (0)	0 (0)	0 (0)	0 (0)	0 (0)	0.593	0.640	0.872	0.456
34. Nuts	5.7 (9.4)	5.7 (10)	4.3 (11.4)	5.7 (12.3)	5.7 (11.4)	11.4 (14.3)	8.6 (8.6)	8.6 (17.1)	0.377	**0.002**	**0.011**	0.182
35. Dairy deserts	0 (42.9)	0 (28.6)	0 (28.6)	0 (57.1)	14.3 (100)	14.3 (42.9)	0 (28.6)	0 (28.6)	0.304	0.327	0.918	0.566
36. Cakes	0 (6.7)	0 (6.7)	0 (6.7)	0 (13.3)	0 (3.3)	0 (3.3)	0 (6.7)	0 (3.3)	0.057	0.120	0.972	0.172
37. Chips	2 (4.3)	2 (4.3)	2 (4.3)	2 (4.3)	2 (4.3)	1 (4.3)	0 (4.3)	0 (2)	0.831	0.612	0.258	**0.018**
38. Candies	0 (1.3)	0 (1.3)	0 (0.7)	0 (2.7)	0 (1.3)	0 (0)	0 (0)	0 (0)	0.763	**0.032**	0.253	**0.002**
39. Ice creams	0 (14.3)	0 (14.3)	0 (14.3)	0 (6.7)	0 (14.3)	0 (14.3)	0 (14.3)	0 (6.7)	0.805	0.568	0.725	0.687
40. Sugar beverages	0 (33.3)	0 (20.8)	0 (35.7)	0 (35.7)	0 (35.7)	0 (35.7)	0 (35.7)	0 (33.3)	0.519	0.681	0.770	0.870
41. Hight beverages	0 (35.7)	0 (53.6)	0 (33.3)	0 (35.7)	0 (35.7)	0 (35.7)	0 (8.3)	0 (16.7)	0.669	0.853	0.830	0.397
42. Wine	0 (3.3)	0 (1.7)	0 (10)	0 (6.7)	0 (14.3)	0 (14.3)	0 (14.3)	0 (6.7)	0.111	0.095	0.344	0.915
43. Beer	0 (6.7)	0 (6.7)	0 (13.3)	0 (13.3)	0 (28.6)	0 (28.6)	0 (28.6)	0 (20)	0.346	0.376	0.326	0.663
44. Free alcohol beer	0 (0)	0 (0)	0 (0)	0 (0)	0 (0)	0 (0)	0 (0)	0 (0)	0.454	**0.040**	0.085	0.058
45. Distilled beverages	0 (0)	0 (0)	0 (0)	0 (0)	0 (0)	0 (0)	0 (0)	0 (0)	0.934	0.414	0.320	0.546

CTR: control; INT: intervention. The number of participants varied across assessments (see Figure 1). Bold fonts stand for significant values.

**Table 6:** Effects of the intervention by group and time for fermented food and polyphenols and omega-3 rich food.

	CTR Group, n (%)				INT Group, n (%)				CTR vs. INT (*p*-Values)			
	0 Month	3 Months	6 Months	12 Months	0 Month	3 Months	6 Months	12 Months	0 m	3 m	6 m	12 m
Fermented food	11 (13.1)	5 (7.7)	7 (13.2)	7 (13.7)	15 (20.8)	26 (42.6)	25 (41)	18 (29.5)	0.196	**<0.001**	**0.001**	**0.025**
Polyphenols rich fruits	28 (33.3)	16 (24.6)	18 (34)	17 (33.3)	17 (23.6)	31 (50.8)	32 (52.5)	24 (39.3)	0.181	**0.002**	**0.025**	0.311
Other polyphenols foods	31 (36.9)	20 (30.8)	20 (37.7)	15 (29.4)	32 (44.4)	23 (37.7)	22 (36.1)	20 (32.8)	0.339	0.412	0.983	0.488
Omega-3 rich food	44 (52.4)	25 (38.5)	22 (41.5)	32 (62.7)	32 (44.4)	43 (70.5)	37 (60.7)	39 (63.9)	0.323	**<0.001**	**0.019**	0.451

CTR: control; INT: intervention. The number of participants varied across assessments (see Figure 1). Bold fonts stand for significant values.

**Table 7:** Effects of the intervention by group and time for 24 h dietary recall (24 HR), chrono-nutritional factors and anthropometrics.

	CTR Group, Median (IQR)				INT Group, Median (IQR)				CTR vs. INT (*p*-Values)			
	0 Month	3 Months	6 Months	12 Months	0 Month	3 Months	6 Months	12 Months	0 m	3 m	6 m	12 m
***24HR items*** Energy (Kcal/day) HC % Fat % Protein % Sugar (g) Fiber (g) Protein (g) Iron (mg) Vitamin B12 (µg) Vitamin C (mg) Mg (mg) ***Chrono-nutrition*** Meals/day (n) Night fasting (h) Night eating, n (%) ***Anthropometrics*** BMI (kg/m^2^) Normal Overweight Obese Waist perimeter (cm) SMMI (Kg/m^2^) Low muscle mass Normal muscle mass	1034.93 (633.2) 42.75 (14.71) 37.73 (13.7) 18.87 (7.03) 29.45 (32.01) 9.38 (10.08) 49.2 (28.15) 5.35 (3.53) 1.82 (1.8) 62.8 (103.96) 138.51 (83.76) 4 (1) 11 (2) 15 (17.85) 28 (10) 24 (27.9) 27 (31.4) 35 (40.7) 98 (24) 6.7 (1.51) 44 (51.2) 42 (48.8)	115.42 (591.26) 42.02 (11.51) 37.31 (12.65) 18.67 (7.26) 35.95 (32.95) 11.75 (10.48) 56.62 (28.73) 6.37 (4.68) 1.7 (2.59) 59.72 (72.14) 168.95 (110.78) 4 (1) 11 (3) 13 (20) 27.6 (8.05) 21 (30.9) 24 (35.3) 23 (33.8) 94.25 (20) 6.68 (1.37) 35 (52.2) 32 (47.8)	122.42 (516.41) 43.25 (11.67) 38.87 (12.18) 17.16 (6.64) 33.5 (27.7) 10.82 (9.97) 52 (25.91) 5.66 (3.96) 2.01 (2.02) 51.76 (84.98) 144.34 (97.3) 4 (1) 11 (2.5) 11 (20.75) 6.8 (8.7) 19 (34.5) 19 (34.5) 17 (30.9) 95 (20.5) 6.68 (1) 29 (53.7) 25 (46.3)	1158.73 (52.6) 43.89 (14.78) 38.61 (11.73) 17.47 (8.26) 39.62 (29.66) 11.79 (11.29) 52.34 (21.6) 4.94 (3.25) 1.37 (1.45) 57.24 (78.88) 153.12 (78.76) 5 (1) 10 (2.5) 13 (25.49) 28.15 (10.85) 18 (34.6) 17 (32.7) 17 (32.7) 92.75 (23.5) 6.55 (1.3) 29 (55.8) 23 (44.2)	1166.12 (464.63) 42.68 (9.6) 38.61 (11.73) 18.83 (5.37) 31.95 (28.5) 9.02 (8.36) 54.47 (18.13) 5.38 (2.71) 2.76 (2.28) 59.36 (71.36) 160.29 (92.28) 5 (1) 11 (2) 14 (19.44) 27.35 (8.45) 22 (30.6) 20 (27.8) 27 (37.5) 95.5 (21) 6.79 (1.4) 34 (47.2) 38 (52.8)	1237.03 (499.58) 40.97 (9.74) 39.3 (10.79) 18.76 (6.17) 35.15 (27.11) 12.54 (11.7) 58.94 (26.16) 7.07 (4.01) 2.77 (3.68) 76.28 (95.75) 170.61 (118.35) 5 (1) 11 (2) 7 (11.47) 27.30 (7.60) 15 (24.6) 21 (34.4) 22 (36.1) 99.25 (21.75) 6.95 (1.53) 28 (45.9) 33 (54.1)	1295.65 (291.35) 41.52 (10.11) 40.24 (7.79) 17.63 (5.76) 33.3 (31.63) 11.78 (9.76) 60.51 (23.95) 6.09 (3.36) 2.41 (3.16) 68.73 (81.43) 181.96 (109.79) 4.75 (1) 11 (2) 8 (13.11) 27.45 (8.60) 14 (23.3) 22 (36.7) 22 (36.7) 95.5 (19.75) 6.81 (1.49) 28 (46.7) 32 (53.3)	1176.46 (453.18) 39.47 (10.68) 39.82 (10.62) 19.56 (7.16) 29.19 (21.07) 10.98 (10.63) 58.79 (26.99) 6.78 (5.02) 2.07 (3.54) 66.1 (89.9) 156.59 (106.63) 4.5 (1) 11 (2) 5 (8.19) 27.20 (8.70) 16 (28.1) 19 (33.3) 21 (36.8) 95 (19.5) 6.88 (1.42) 22 (38.6) 35 (61.4)	0.660 0.514 0.341 0.788 0.353 0.916 0.370 0.386 **0.009** 0.804 **0.045** 0.891 0.344 0.799 0.461 0.715 0.620 0.682 0.918 0.883 0.622 0.622	0.643 0.689 0.525 0.682 0.796 0.246 0.158 0.175 **0.004** 0.101 0.299 0.230 0.092 0.191 0.944 0.426 0.918 0.790 0.519 0.461 0.474 0.474	0.187 0.211 0.067 0.350 0.839 0.285 0.132 0.382 0.164 0.280 0.075 0.299 0.455 0.331 0.549 0.184 0.812 0.515 0.294 0.592 0.453 0.453	0.427 **0.036** 0.159 0.193 0.088 0.691 0.056 **0.014** **0.001** 0.869 0.242 0.220 **0.016** **0.022** 0.983 0.461 0.943 0.650 0.403 0.350 **0.073** **0.073**

The number of participants varied across assessments (see Figure 1). Bold fonts stand for significant values.

## Data Availability

The datasets presented in this article are not readily available because the data are part of an ongoing study. Requests to access the datasets should be directed to the corresponding author.
